# The meanings of cancer and perceptions of cancer services among South Asians in Luton, UK

**DOI:** 10.1038/sj.bjc.6601892

**Published:** 2004-05-25

**Authors:** G Randhawa, A Owens

**Affiliations:** 1 Institute for Health Services Research, University of Luton, St Nicholas House, 15-17 George Street, Luton LU1 2AF, UK; 2 Department of Geography, Queen Mary, University of London, Mile End Road, London E1 4NS, UK

**Keywords:** cancer awareness, cancer services, South Asian, cultural competence

## Abstract

Recent research has suggested that there is limited awareness of and information about cancer and cancer services among South Asian communities. This study explores the meanings of cancer and perceptions of cancer services among South Asians living in Luton. Six single-sex focus groups were conducted among the three main South Asian groups in Luton: (1) Punjabi-speaking Muslims originating from Pakistan (Pakistani Punjabi); (2) Sylheti-speaking Muslims originating from Bangladesh (Bangladeshi Sylheti); and (3) Punjabi-speaking Sikhs originating from the Indian Punjab (Indian Punjabi). Overall, it was found that the information relating to cancer for South Asian communities was limited. Participants in the study expressed a keen desire for this information to be made available via their community social networks. This lack of information resulted in low levels of awareness about cancer and related issues. Cancer was often perceived as an incurable disease, a reflection of the fact that access to appropriate services had been experienced at a relatively late stage of the illness. Informed education, therefore, is clearly essential to influence how people manage cancer and access cancer services. This paper describes the challenges that service providers and users face in ensuring effective and informed awareness.

Over the last 20 years, there has been a growing interest in the health of minority ethnic populations in the UK. In 1991, for the first time, the UK census included a question on ethnic identity. The information obtained from this question has enabled the health status of different ethnic groups to be investigated more systematically. These investigations revealed a number of pressing health concerns among minority ethnic groups that need to be addressed. Government strategy papers such as the *Health of the Nation* and the more recent *Our Healthier Nation* have highlighted some of these specific health problems and the need for an urgent course of action to reduce health inequalities (DOH, 1992, 1999). In 1994, The Department of Health established the NHS Ethnic Health Unit, which aimed to promote health and health care for minority ethnic groups. The Health Education Authority has carried out two health and lifestyle surveys of black and minority groups, which have sought to highlight information regarding health behaviours and health status (Rudat, 1994). The Fourth National Survey, conducted by the Policy Studies Institute (PSI) and Social and Community Planning Research (SCPR), included an extensive section on health among minority ethnic groups (Nazroo, 1997). Most recently, the seminal *Tackling Health Inequalities Report: A Programme for Action* published by the Government has revitalised national interest in reducing inequalities (DoH, 2003).

Throughout this period, the provision of coronary heart disease, mental health and diabetes services for minority ethnic groups have become particularly important areas of concern. This is in part due to the observation of high rates of these conditions within particular minority ethnic groups. However, it is only relatively recently that the provision of appropriate cancer and palliative care services for minority ethnic groups has begun to be substantively explored. An important explanation for this policy lag is that cancer has not been regarded as a major medical problem among many minority ethnic communities. However, recent epidemiological evidence demonstrates that lifestyles are changing and that cancer is becoming a more significant problem among such communities. As the needs of particular minority ethnic groups become clearer, questions relating to the nature and scope of cancer and palliative care services have moved to the forefront of the minority ethnic health care agenda. As a landmark policy development in the provision of cancer services, the NHS Cancer Plan is, for example, particularly attentive to providing services and information that can meet the needs of minority ethnic groups, such as in the area of screening and palliative care (DoH, 2000a, 2000b, pp 40–41, 68). A renewed concern for equity of health care provision, coupled with a growing understanding of cancer among minority ethnic groups therefore poses new challenges for service providers in terms of ensuring that Britain's minority ethnic populations understand what cancer is and the risk factors that can cause it. Equally, services must be developed that can be easily accessed by minority ethnic groups and that can meet their needs in a ‘culturally sensitive’ way. In order to do this, it is necessary to know something about current levels of awareness and understanding about cancer among minority ethnic groups. A recent report by the National Cancer Alliance has suggested that ‘there is limited research available which considers the information needs of South Asian cancer patients and their carers’ (National Cancer Alliance, 2002, p 3). This article contributes to redressing this omission for one of the UK's most significant minority ethnic populations.

The study reported within this article aims to support this effort by examining the views of South Asians in Luton towards cancer and cancer services. The term South Asian here applies to people originating from the Indian subcontinent. The article begins by setting out current understanding on the prevalence of cancer among South Asian people. It then outlines the qualitative methods used in this study, before presenting results of focus group discussions carried out with South Asian community representatives. Finally, we draw some conclusions from the study and highlight potential policy developments aimed at increasing knowledge, awareness and uptake of cancer services among South Asian communities, within the policy formation context of the NHS Cancer Plan.

## CANCER AND SOUTH ASIANS IN BRITAIN

Recent epidemiological studies of the prevalence of cancer among Britain's South Asian populations have generally concluded that rates of cancer are considerably lower among such communities than among the population as a whole. The pioneering comparative study by Donaldson and Clayton (1984) of cancer in Asians and non-Asians in Leicester demonstrated standardised registration ratios (SRRs) for breast, trachea bronchus and lung, colon and rectum, and secondary (respiratory and digestive) cancers, and for leukaemia that were lower among the Asian communities than the population as a whole. (The SRR is a measure of the registered incidence of different types of cancer relative to the standard population and adjusting for the age structure of the group being studied.) However, they also noted a higher incidence of oral and oesophageal cancers. Since publication of these data, a significant number of other studies have examined the prevalence of cancer among South Asian populations or specific subgroups, such as Britain's Bangladeshi communities (Marmot *et al*, 1984; Balarajan and Bulusu, 1990; Balarajan and Raleigh, 1998; for a review of studies, see Bhopal and Rankin, 1996). In general, these studies confirm the findings of Donaldson and Clayton (1984) in suggesting that rates of cancer are often significantly lower among South Asian communities than among the population as a whole. The evidence provided by Barker and Baker (1990), for example, pointed to a lower occurrence of cancers of the stomach, bowel, lung, skin and bladder in South Asian male population, and reduced cases of skin, breast, cervix and ovary cancers in South Asian female population. However, these studies are also united in suggesting that for specific sites cancers are more common among South Asian populations. Cancers of the gall bladder and liver have been demonstrated to be higher. Similarly, a relatively high occurrence of various mouth cancers has been demonstrated and a number of authors have suggested that this relates to the popularity of paan, betel-quid or tobacco chewing among some Asian populations (Bedi, 1996; HEA, 1996, 2000). Small-scale studies conducted by Healy and Aslam (1989) support this view and state that the custom of chewing paan is so widespread among the Bangladeshi community in Britain that the incidence of oral cancer within this group appears to be on the increase. Indeed, recent research carried out among Britain's Bangladeshi communities has sought to heighten attention to the risks of cancer that result from paan chewing (Bedi and Jones, 1995) This in one area where health promotion initiatives have been focused on South Asian communities.

Barker and Baker (1990) concluded that the decreased incidence of cancer among South Asians may be due to a lower exposure to risk factors. However, their study was also keen to point out that the situation was a changing one. Comparable data were used to show that cancer rates among their study group of Bradford ‘Asians’ were higher than those among the ‘home country’ populations of Mumbai (Bombay), suggesting that changes in environment and lifestyle were impacting on cancer morbidity. (Obviously, not all of Bradford's Asian population are of Mumbain (Bombayan) or even Indian origin. Donaldson and Clayton (1984) use Mumbai (Bombay) merely as a proxy for a ‘home’ population.) They also found evidence that within Bradford, rates of certain types of cancer, such as breast cancer, were increasing. This suggestion is supported by a more recent and comprehensive study of cancer incidence among England's South Asian populations carried out by Winter *et al* (1999), which attempted to look at the differences between populations of South Asian ethnicity born in England and those of south Asian ethnicity born in the Indian subcontinent. Drawing upon 356,555 patient records in the Thames, Trent, West Midlands and Yorkshire cancer registries over the period 1990–1992, they came to the familiar conclusion that incidence rates among English South Asians were ‘significantly lower’ than non-South Asians. However, they also discovered that the rates among English South Asians were ‘significantly higher’ than among those South Asian immigrants born in the Indian subcontinent.

Overall, therefore, while the evidence generally does suggest that cancer morbidity and mortality rates among South Asian populations are lower than average, it would appear that where differences do exist, they are diminishing and cancer rates are moving closer towards those of the mainstream population. This ‘epidemiological transition’ is argued to be occurring as lifestyles change and ‘hybridise’, shifting closer to those of the mainstream population, particularly among the second and third generations of immigrants (Smaje, 1995, p 63; Winter *et al*, 1999). As a result, it is no longer possible to agree with Karmi and McKeigue (1993) that cancer ‘is not especially relevant to ethnic groups in the UK’. Indeed, this epidemiological evidence suggests an urgent need to ensure that South Asian people have a good understanding of cancer and the risk factors that can cause it, along with an ability to access cancer services that are attuned to their needs. At present, evidence suggests that South Asian patients are not accessing cancer services as much as other groups of the population. Raja-Jones (1999), for example, demonstrates that uptake of breast screening remains low in South Asian communities, while a recent survey for the Health Education Authority (2000) showed that a high proportion of South Asian women between the ages of 16 and 74 had not had a cervical smear test. In the field of palliative care, numerous studies have demonstrated a low take up of services by Britain's South Asian populations (for a review, see Randhawa and Owens, 2004).

This article presents the findings of an exploratory study, commissioned by Luton Health Action Zone that investigated access and use of cancer services among South Asians in Luton. South Asians make up 18.3% of the population. Health Action Zones were established by the UK Government in 1998 to tackle the wider determinants of health, such as ethnic background, poverty, housing and transport, in the areas of worst health in the UK. In keeping with this agenda, a principal aim of the study was to explore the awareness and attitudes towards cancer and cancer services, in order to improve services so that they become more accessible.

## METHODS

A qualitative study was undertaken, which involved setting up focus group discussions with a cross-section of Luton's South Asian populations in order to assess knowledge of and attitudes towards cancer. Cancer, being particularly associated with death, is a very sensitive subject and not a common topic for conversation or discussion. Thus, the use of focus groups was seen as integral to the research process as they have been shown to actively facilitate the discussion of taboo topics such as bereavement and sexual violence because the less inhibited members of the group can break the ice for shyer participants (Kitzinger, 1995). In-depth interviews were also carried out with professionals who worked in the cancer and palliative care field in order to gain their perspectives on cancer and cancer service awareness within local South Asian communities. This research methodology was developed in consultation with and eventually approved by the local medical research ethics committee.

The study sample of non-professionals was selected on the basis of the geographic origin, language spoken and religion in order to reflect the demographic profile of the Luton's South Asian population. Four key groups of participants were originally identified:

- Gujarati-speaking Hindus originating from India (Indian Gujarati);
- Punjabi-speaking Sikhs originating from the Indian Punjab (Indian Punjabi);
- Punjabi-speaking Muslims originating from Pakistan (Pakistani Punjabi);
- Sylheti-speaking Muslims originating from Bangladesh (Bangladeshi Sylheti).

In line with other research carried out among South Asian communities, single-sex focus groups were selected in order to encourage freer dialogue and to enable consideration of gender-specific perspectives. In total, therefore, eight focus group discussions were planned, comprising one male and one female group for each of the four identified community strands. Unfortunately, due to organisational difficulties and the pressure of the project timetable, it became impossible to arrange the focus groups with the Hindu Gujarati community.

As Fuller *et al* (1993) state, ‘focus groups are rarely, if ever, composed of random samples of a target population’. Indeed, no claim can be made that the focus groups selected in this study are representative of a broader population in any statistical sense. However, the groups did provide access to a broad cross-section of the South Asian communities in Luton. There were 6–10 people in each group, with a total of 48 people participating in the discussions. The groups contained people of a range of ages and were recruited through community contacts and were conducted at neutral venues to promote freer discussion. All of the focus group discussions were conducted in the appropriate South Asian language. Three of the research team were fluent in either Urdu and Punjabi and were able to act as moderators for some of the focus groups. Additional moderators, who had prior training in other research studies, were recruited to undertake the Gujarati and Sylheti focus groups. As Rehman and Walker (1995) note, conducting fieldwork among South Asian origin populations not only needs moderators fluent in the mother tongue of that population but also people who are aware of cultural nuances in order to communicate effectively and interpret data. It is often thought that the use of the mother tongue is necessary only for older members of the population. However, many young people fluent in English are bilingual and switch from one language to another with ease and often utilise their ethnic language or words from it to convey meaning about concepts or situations alien to white culture. As the group discussions included people ranging from 18 to over 60 years, it was ensured that all moderators were bilingual.

Given the relative dearth of studies considering the provision of cancer services to minority ethnic groups, there were few pretested questions available with which to devise a topic guide for the focus groups. The content and structure of the guide stemmed from a study of the available literature and evolved through a series of discussions among the researchers and the project steering group. The final topic guide covered the following themes: understandings of cancer; experiences of cancer; knowledge of cancer risk factors; knowledge of cancer services in Luton; and sources of cancer-related information. Care was taken to translate the guide so that the meaning of different themes and topics was not distorted. The topic guides were piloted in different languages by each of the moderators. After this process, some of the questions were rephrased to make their understanding clearer.

A series of semistructured, in-depth interviews were carried out with professionals who work in the cancer and palliative care field in Luton. Respondents were chosen with an overall aim of trying to gain the opinions of a broad plethora of palliative care workers from majority and minority ethnic backgrounds. A sample of 10 interviewees were selected, principally from those who work in community settings. The sample included community nurses (both general, specialist and those affiliated to specific organisations, such as the Macmillan service), community health liaison personnel and representatives of voluntary organisations who provide care services for families with terminally ill members. In addition, a member of staff at the local hospice and a specialist palliative care nurse based at the local hospital were also interviewed; both had previous experience of community nursing.

Analysis of the focus group discussion and interview transcripts was undertaken in a similar manner to the technique of O'Brien (1993). The transcripts were read by the researchers and particular words or phrases used to describe experiences were listed and grouped together on the basis of similarity to highlight emerging categories. The categories were summarised and listed together and then grouped into themes, based on their similarity in content. These themes were re-examined and, where appropriate broken down into subthemes. The themes were then used to analyse the transcripts of all of the focus group discussions in order to determine and reflect upon the range of views and experiences considered.

## LIMITATIONS OF THE STUDY

As described in the methodology section, the aim of the study was to carry out an exploratory study that provided a ‘snapshot’ of current levels awareness towards cancer and its risk factors. Participants for the focus groups among the South Asian communities were recruited from a broad age range to reflect a cross-section of the South Asian communities in Luton. The authors acknowledge that this recruitment approach has limitations in terms of generalisability of findings to the wider South Asian population of the UK. Future research in this area would benefit from larger study samples that ascertain information on potential confounding variables such as educational background, length of residence in the UK and languages spoken. This would enable interesting comparisons between the different South Asian communities of similarities and differences of their perceptions towards cancer and its risk factors.

## RESULTS

Naturally, the conclusions drawn from the analysis that follows apply to this sample alone and cannot be generalised in a straightforward manner to the wider UK South Asian population. As the study involves small and statistically unrepresentative samples, elaborate statistical analysis has not been attempted. Nonetheless, this approach highlights themes and trends that allow for some speculation about the wider populations at large. We have chosen to quote extensively from the transcripts rather than paraphrasing responses in our own words. This has been undertaken in order to try and provide a sense of the context in which things were said and in order to preserve some of the nuances and more subtle meanings of speech that might have been lost through paraphrasing. The article focuses on four key themes: knowledge of cancer, experiences of cancer, understandings of the causes of cancer, and awareness of cancer services and associated information needs.

### Knowledge of cancer

One of the central aims of the study was to establish a baseline of knowledge within the South Asian community concerning cancer and cancer services in Luton. The British Medical Association have provided a lay definition of cancer as, ‘any of a group of diseases in which symptoms are due to the unrestrained growth of cells in one of the body organs or tissues’. The focus group participants were asked whether they knew what cancer was and to illustrate this by explaining what they understood cancer to be. Only two of the 48 respondents had not heard of cancer, which indicated that although people may not necessarily have been able to define exactly what cancer was as an illness, there was, at the very least, an awareness of cancer as some form of illness.

Responses to participants' understanding of what cancer was as an illness fell into two distinct themes: an incurable disease and a frequently cursed – ‘nasty’ and ‘horrible’ – serious illness (see Box 1

**Box 1:** Definitions of cancer

*I think it is a nasty disease.*

*Rotten thing. Sometimes it's very very – it's decaying thing.*

*This is a very serious illness.*

*Cancer is something, there is no medicine out there, there is no cure for it.*

*Some sort of virus which no medicine can kill those virus and kill all system.*

*Cancer is dangerous disease which is broken blood cell, is not curable later.*

for some illustrative quotes). (Throughout the article, all quotes have been reproduced verbatim and have not been corrected for grammatical reasons.) As Chattoo *et al* (2002) have suggested, such understandings are not just confined to South Asian patients but are common across different ethnic groups. The emotive language used to describe the disease signifies, as they suggest, ‘the stigma, bleakness and hopelessness associated with cancer’ (Chattoo *et al*, 2002, p 12). This was a point that was reinforced through the interviews with health care professionals who harboured a pervasive fatalistic attitude towards cancer among their South Asian patients. This understanding of cancer as frequently being terminal may be indicative of the late or low uptake of cancer services that is apparent in South Asian communities.

Overall, knowledge of cancer varied among the participants and there were distinct differences between the focus groups, suggesting that it is inappropriate to generalise about knowledge and awareness too much. The meaning of cancer to participants in the study was also frequently articulated at a more physical and practical level, such as in terms of its symptoms. ‘It gives you hair loss’ was a typical remark. Indeed, some responses disclosed a greater understanding of the disease, discerning different types of cancer, the possibility that some might respond to treatment, and an awareness of the temporal dimensions of the illness.

I know there are a lot of different forms of cancer. I know there are some that can be treated if they are found early on, in the body. I think it also depends on your age.Maybe some curable early stages, like breast cancer is curable, like mouth cancer is curable, but some things – blood cancer, like if it's children blood cancer is curable, when they adult stage is not curable. Bone cancer, early stage is curable but not later and what other things – stomach cancer, bowel cancer…

As a follow-up to this question, the participants were asked about where they had ascertained their information concerning cancer. Nearly all of the respondents stated that they had learnt about cancer through either family or community networks. This was especially the case for the Bangladeshi and Pakistani respondents. Within the Sikh focus groups, some respondents said they knew of cancer through their GP, while others suggested their knowledge of cancer was shaped by television and newspapers. The information broadcast via TV and in the newspaper was received in English. One respondent, a Bangladeshi male, made reference to information he received via a leaflet that had been translated into an Asian language. He had picked up this leaflet in an area of London where there was a large resident Bangladeshi population.

In some cases, it was clear that focus group participants saw cancer as something of a taboo – an ‘unspeakable disease’ as Chattoo *et al* (2002, p 12) put it – and therefore had avoided trying to seek information about it. However, there was also an acknowledgement that efforts could be made to find out about cancer if the need arose. The Sikh women's focus suggested that they would look for information concerning cancer in the newspaper and on television in English if the need arose. However, they expressed a need for leaflets to be produced in Punjabi as they felt these would be more accessible. There was also a desire to have more information provided about cancer on the Asian television channels. The potential of television and related visual media to inform South Asian people about cancer and cancer services has been noted by other researchers (National Cancer Alliance, 2002, p 36).

### Experiences of cancer

The focus group discussions moved on to explore the participants' experiences of cancer. Interestingly, the majority of Pakistani respondents knew of people, either within their family network or community network, who had suffered from cancer. Two of these respondents made reference to the local hospital providing some input to cancer care. None of the Bangladeshi men were aware of anyone who had cancer. Two of the Bangladeshi women, however, knew of people who suffered from the illness. Among the Sikh respondents, only three people knew of someone who had cancer (see Box 2

**Box 2:** Experience of cancer

*We haven't anyone in the family but I know someone who has cancer, their daughter has cancer. So we hear about it that it is very painful. We also knew someone in Pakistan who had three or four children who all died from cancer. Thirteen fourteen years of age, one five year old. Didn't realise they had cancer. When it was in its final stage, they found it.*

*One of my close friends, actually she went on holiday to Pakistan. When she came back she had a very high temperature. When she got back within a week she was admitted into hospital, because of the temperature and she was expecting as well. They couldn't figure out why her temperature was so high. She had advance cancer of the throat. The only time it comes up, that type of cancer, the only time they can actually diagnose it is in its advanced stage, and you can't do anything about it. Within four weeks she died.*

*Even with the young person died. The treatment might have lacked in something. There should be some research done, and to concentrate on it. The child had his treatment done, he stayed alive for six years. He had started a job. After that when he was twenty-six years of age the cancer came back and then he died.*

*Have experience, but, they were related to us, like I said they had hair loss, they didn't know that they had cancer. The doctor said, they didn't go to the doctors, kept taking paracetamol. Her condition got worse, they got admitted into hospital, they were told then it was cancer. They couldn't do anything.*

*We never told our father he had cancer. He was very ill first, few days how long he survived.*
What, the family never told him?
*The family, yes. He didn't know it. This is the last because there is no cure.*

for some illustrative quotes). While the numbers of people involved in the focus groups mean that generalisations cannot be made, it is clear that cancer is an illness that figures in the lifeworlds and social networks of South Asian people and therefore disputing any view that cancer incidence is low and ‘not especially relevant’ (Karmi and McKeigue, 1993) to minority ethnic populations. This was also a view that did not accord with the experiences of health care professionals working with Asian populations.

What is striking about these accounts is the way that virtually all respondents had encountered cancer at a late stage of the illness, that the patient had not realised that he or she had cancer, and that it had resulted in death. This perhaps partly explains why many respondents earlier defined cancer as an incurable disease and talked about it in a fatalistic way. While the terminal rather than the early phases of cancer are likely to be the time when people ‘notice’ the disease, that experiences of cancer are often so late on in the illness among South Asian people, is cause for concern. This underlines the fact that more information and awareness would not only result in a better understanding of the disease that demonstrates curative treatment can sometimes be an alternative to palliation, but may also result in cancer sufferers presenting themselves to services at an earlier stage in the illness.

### Causes of cancer

The focus group participants were asked whether they knew about the causes of cancer. Nearly all of the respondents were aware that smoking was linked to cancer. Once again this echoes other research, where health practitioners expressed concern that the effectiveness of health promotion messages about the links between smoking and cancer was obscuring and diverting attention from other risk factors (National Cancer Alliance, 2002, p 13). The Sikh men also made reference to passive smoking being a potential risk factor. The Bangladeshi and Pakistani respondents were all aware of the link between chewing paan and cancer, reflecting perhaps, as was noted earlier, the success of specific awareness campaigns in relation to this issue. A number of respondents also made reference to the possible links between diet and cancer (see Box 3

**Box 3:** Causes of cancer

*I have heard smoking causes cancer.*

*Some say it's from the cigarette. Why men got it. The women don't smoke but they get it?*

*It's from God why it happens to some people. It's from God willing because some people don't smoke, because sometimes it happens in the lung, sometimes it happens in the lung, or bone, or blood.*

*The people who eat betel leaf can cause it.*

*Lack of calcium in your diet causes bone cancer, and I am worried because I always have pains and aches in my bones.*

*Someone told me that eating too much ghee causes cancer of the stomach.*

*We should we care about what we eat.*

for some illustrative quotes).

On a positive note, the above quotes suggest that there is a good level of awareness of potential risk factors for cancer. The key challenge, therefore, appears to be modifying behaviour in the South Asian population in reducing their exposure to risk factors.

### Cancer services in Luton

Another key element of the focus group discussions was to establish people's understandings of cancer services and how they could be accessed. This included a consideration of where people said they would go if they had particular concerns related to cancer and whether they were aware of any specialist cancer services in Luton. All of the respondents stated that they would go to see their GP first concerning any health issue, cancer related or not. Awareness of more specialist cancer services in Luton was concerningly low. Some of the Sikh women were aware of screening services such as smear tests and breast screening. Three people knew of the hospice, but no one in the focus groups was aware of the term ‘palliative care’, or services, such as the Macmillan nursing care, that local palliative care provision included. Health care professionals confirmed a lack of awareness and understanding of cancer services among South Asian populations, as the following comments about the local hospice illustrate:

There don't seem to be many (South Asian Patients) attending there and I've just spoken to one and she's got no idea what it's about’ that's why she wouldn't go. But when we actually got her to go, she started attending and she really likes it … I don't think they realise that the service is available and the fact of what it does.One came in, was only last week actually, and was – they had the – the hospice to them was quite a frightening area to come to. And the patient that went home last week was surprised what a different place it was to what she had actually imagined.

Many of the professionals interviewed suggested that the low visibility of South Asian patients within cancer and palliative care services reflected the fact that when services were offered, they were often refused. This clearly troubled some respondents:

On a personal level I find it very difficult when you know these services are available and you're trying to promote them, and people don't want to take them on board …It's very hard, because you can see an instance where someone could so benefit – I'm talking about the hospice here.

However, professionals also suggested, however, that levels of awareness and receptiveness to services varied with age – younger people had a greater knowledge and were more willing to find out what was on offer.

Given this low level of awareness of cancer services in Luton, the focus group respondents were asked for their opinions on how best to provide information about such services to the South Asian populations of the Borough. The overwhelming suggestion was that the information be provided in the various South Asian languages, and that it ought to be channelled through community networks using seminars, or existing forums (see Box 4

**Box 4:** Cancer services in Luton

*First thing is that, there are Punjabi speaking. Information from the very beginning the cancer illness, its cure, all this information. Think about what facilities the area needs, there should something in all these areas. And those leaflets, like in our Gurdwara, so that any person can pick them up and read them.*

*A group discussion would be better….in my language.*

*If you do have a service open to the general public, especially to the Asian community, there should be interpreters available to interpret the information to that person, rather than having a service and when you go there you're just given a lip-service and told this and that.*

*Especially pictures of young children, Asian women, you know different snap shots ands say well ‘What do all these people have in common?’ Obviously are going to relate to one of them, or two of them. And then when you say well they all suffer from cancer and then they will taken interest. Um whereas lot of time, it is a lot, very hidden, you have to look for it yourself.*

*I think it has to be a lot more user-friendly.*

for some illustrative quotes). Similar to the findings of the National Cancer Alliance (2002, p 19) study, comments suggested that information should be both introductory and detailed as well as being tailored to individual circumstances and social structures. Professionals were sympathetic to and actively trying to pursue such suggestions. A number of interviewees pointed to the importance of an individualised approach: building up a rapport with clients and their families and of being persistent in offering services to them. More general initiatives aimed at improving cancer service awareness among Luton's Asian populations, such as a health roadshow, were considered not to have been successful partly because of the taboos attached to the disease.

## DISCUSSION AND CONCLUSION

This exploratory study has provided a ‘snapshot’ picture of the level of awareness to and perceptions of cancer and cancer services among South Asians in Luton. It has also illustrated some of the ideas and suggestions that South Asian people have on how to improve services so they become more accessible for them. The focus group discussion revealed a low level of awareness of cancer and cancer services among Luton's South Asian populations. An over-riding concern expressed in all the groups was the lack of available information relating to cancer and available health care services. The interviewees expressed a keen desire for this information to be made available via their community social networks. The current lack of information is clearly resulting in ignorance and may be leading to unnecessary suffering and death. Cancer was frequently perceived as incurable disease, as access to appropriate services had been experienced at a late stage of the illness. Informed education, therefore, is clearly essential to influence how people manage cancer and access cancer services.

As has been noted throughout, many of the findings reported upon in this study chime with research published in the ‘grey literature’ that has sought to understand why cancer services appear to be accessed less frequently by South Asian people than the majority population (Chattoo *et al*, 2002; National Cancer Alliance, 2002). They also perhaps reflect a wider problem that health professionals face in providing effective health care to minority ethnic populations. Alexander, for example, has recently observed that often people from minority ethnic groups may be discouraged from using health services because of a lack of confidence in the system, a shortage of appropriate information, insensitivity to cultural or social needs, stereotyping or previous negative experiences within it (DoH, 2000a). As Nazroo (1997) notes, where such practice exists, it can not only damage individual patients but can also create and sustain mistrust among the minority ethnic communities as a whole.

So how can cancer services in the community be developed to address some of these issues? At a broad level, a recognition of the social context to health and illness could be important in improving access to services for minority ethnic groups. Helman (1998), for example, has outlined the ways in which a ‘social model of health’ could usefully replace the all-pervasive ‘bio-medical model of health’ that dominates the professional culture of the NHS. Such a model of health, he argues, would enable a much better understanding of the health needs of minority ethnic groups and allow a more sensitive and effective provision of services. With respect to cancer, for example, Helman's model demonstrates the need for minority ethnic groups' understanding of the illness and attitudes to services to be seen within the context of emotional and religious beliefs.

At a more specific level, it is widely recognised that a number of service developments and innovations are required in order to make community health services in general and cancer services in particular more responsive to the needs of minority ethnic groups. Communication has been identified as the key and most crucial factor in all the research conducted around the issues of cancer and ethnic minority groups, whether in the context of preventative health, screening programmes, services in the community, or treatment and care provided in secondary and tertiary settings (see, for example, Somerville, 1998; Randhawa *et al*, 2003).

Cultural ‘insensitivity’ also features high among the factors that maintain inequalities in health and prevent people from minority ethnic groups accessing the services currently available in the UK. It is now widely accepted that ‘cultural competency’ should be an integral part of the training of the health care work force and a central feature of professional practice. While service providers are actively developing a ‘culturally competent’ service in Luton (see Owens and Randhawa, forthcoming for a discussion), they acknowledge that further innovations are necessary to improve awareness and understanding of cancer and cancer services among South Asian people in the Borough. The employment of bilingual linkworkers and efforts to develop a community health care workforce drawn from minority ethnic backgrounds would particularly assist in developing a culturally competent service. The NHS Cancer Plan recommends that in order to further develop cancer services in the community, services need to be sensitive to the needs of the minority ethnic groups and promote greater awareness of the health risks of cancer (DoH, 2000b). Minority ethnic groups' needs should be considered especially in relation to assessment, resource allocation, health care planning and provision.

The NHS Cancer Plan provides an important context within which such service development is currently being considered, developed and implemented. It sets out a very comprehensive national cancer programme for England and summarises recent progress in the areas of cancer prevention, research and improved access. It also outlines an agenda for reducing cancer incidence throughout the entire population. In terms of service provision, it suggests new professional partnerships to improve cancer services in the community, based on more family doctors and community-based nurses, promoting early detection and referral systems (DoH, 2000b). Indeed, the report is keen to suggest that new investment alone is not enough, and that new approaches to address inequalities in cancer incidence and service provision need to be devised. Here the report is sympathetic to the situation that many minority ethnic patients face. It suggests that language, culture and communication barriers need to be addressed in order to reduce inequalities. In their summary, both reports have recommended that training more bilingual patient advocates in cancer-specific areas and inclusion of counselling services are the vital key to success, if people from minority ethnic groups are to make more use of cancer services.

In this broad policy context, several initiatives have been planned and many Health Authorities and other providers have made a positive contribution by developing small-scale strategies within their areas to improve access to cancer services for minority ethnic groups. In Bradford, for example, a palliative care ethnic minority liaison officer has been appointed to promote services to South Asian communities and to improve rates of access and referral (Jack *et al*, 2001). Bradford Community Health Trust has also made concerted efforts to recruit staff from minority ethnic backgrounds in order to promote greater confidence in services among South Asian groups (Daddy and Clegg, 2001). An imaginative project in Birmingham – CAPACITY – funded by the New Opportunities Fund, provides trained community advocates with language skills to provide support for minority ethnic people with cancer. However, some Health Authorities and providers have been more proactive than others. It is therefore clear that consideration needs to be given as to how best to develop small-scale, locally based initiatives for improving minority ethnic health in the cancer arena. Policymakers, clinicians and managers in Luton are endeavouring to take this challenge forward by learning from the views of its South Asian population.
